# Dysregulated liver lipid metabolism and innate immunity associated with hepatic steatosis in neonatal BBdp rats and NOD mice

**DOI:** 10.1038/s41598-019-51143-7

**Published:** 2019-10-10

**Authors:** D. Serrano, J. A. Crookshank, B. S. Morgan, R. W. Mueller, M.-F. Paré, L. Marandi, P. Poussier, F. W. Scott

**Affiliations:** 1 Chronic Disease Program, Ottawa Hospital Research Institute, Ottawa, Ontario Canada; 2 Department of Pathology and Laboratory Medicine, Faculty of Medicine, University of Ottawa, Ottawa, Ontario Canada; 3 Sunnybrook Research Institute, Toronto, Ontario Canada; 4 Department of Medicine, University of Ottawa, Ottawa, Ontario Canada; 5 Department of Biochemistry, Microbiology and Immunology, University of Ottawa, Ottawa, Ontario Canada

**Keywords:** Autoimmunity, Pre-diabetes

## Abstract

In a previous study we reported that prediabetic rats have a unique gene signature that was apparent even in neonates. Several of the changes we observed, including enhanced expression of pro-inflammatory genes and dysregulated UPR and metabolism genes were first observed in the liver followed by the pancreas. In the present study we investigated further early changes in hepatic innate immunity and metabolism in two models of type 1 diabetes (T1D), the BBdp rat and NOD mouse. There was a striking increase in lipid deposits in liver, particularly in neonatal BBdp rats, with a less striking but significant increase in neonatal NOD mice in association with dysregulated expression of lipid metabolism genes. This was associated with a decreased number of extramedullary hematopoietic clusters as well as CD68^+^ macrophages in the liver of both models. In addition, PPARɣ and phosphorylated AMPKα protein were decreased in neonatal BBdp rats. BBdp rats displayed decreased expression of antimicrobial genes in neonates and decreased M2 genes at 30 days. This suggests hepatic steatosis could be a common early feature in development of T1D that impacts metabolic homeostasis and tolerogenic phenotype in the prediabetic liver.

## Introduction

Type 1 diabetes (T1D) is a multi-system disease involving the pancreas, gut, diet and microbial agents^1,2^. We recently identified a gene signature in the pancreas of very young diabetes-prone BB rats^3^. Surprisingly, several of the genes identified in pancreas were also different in the liver, with some hepatic gene changes preceding those observed in pancreas. The liver is the central mediator of metabolism and plays a major role defending against pathogens, in part attributable to its large collection of phagocytes^4^. Although the role of liver metabolism in glucoregulation is well-recognized, it has not been linked to early development of T1D until recently^3^.

There is evidence from prospective studies in humans that dysregulated lipid metabolism precedes the appearance of islet autoantibodies in infants as young as 3 months^5^. There may also be early defects that are reflected in β-cell dysfunction before signs of decreased β-cell mass^6^ that are evident in altered cord blood gene expression^7,8^. The potential dysregulation in the target tissue very early in life suggests it is of developmental origin^7^. Our previous findings in the pancreas of very young diabetes-prone BBdp rats support this conclusion and similar prediabetic changes in liver suggest a role for this organ in diabetogenesis^3^.

The liver shares a common embryonic origin with islets and produces peptides such as SerpinB1, hepatic growth factor (HGF) and IGFBP1 that regulate β-cell function and/or mass^9^. The large concentration of gut-derived molecules that enter the liver via the portal vein requires a robust immune response to pathogens while maintaining a state of unresponsiveness to harmless dietary components and commensal microbes. Any change in this finely tuned balance could play a significant and previously unappreciated role in the pathogenesis of T1D.

In the present study we further investigated changes in hepatic immunity and metabolism in very young BBdp rats compared with control BBc rats and also in NOD mice compared with C57BL/6J control mice. The overall findings indicated that in addition to disrupted metabolism and ER stress in the pancreas^3^, very young BBdp rats demonstrated deficiencies in hepatic macrophages and increased proinflammatory cytokines, suggesting the usual tolerogenic state was impaired. This was accompanied by hepatic steatosis. Similarly, NOD mice had decreased numbers of liver macrophages and increased lipid accumulation in the neonatal period, followed by upregulated UPR gene expression in the pancreas at 30 days. These findings indicate that a more detailed investigation of metabolic dysregulation in liver in T1D pathogenesis is warranted.

## Results

### Fewer hepatic CD68^+^ cells and impaired extramedullary hematopoiesis in BBdp neonates

**Figure 1:**
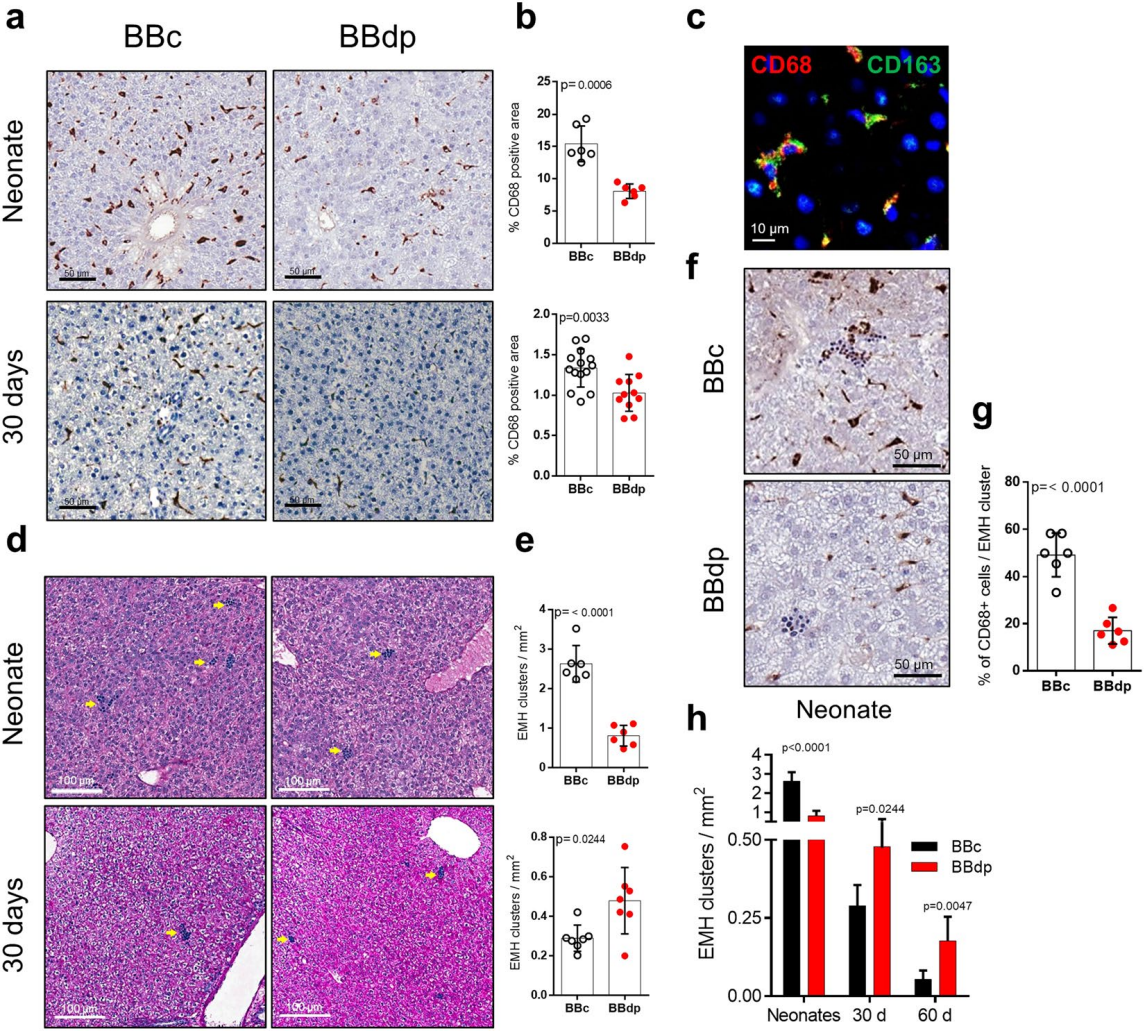
Macrophages and EMH are decreased in neonatal livers from BBdp rats. (**a**) Neonatal and 30 day livers from BBc and BBdp rats were labelled with anti-CD68 antibodies. (**b**) Quantification of the CD68^+^ area in the liver. (**c**) Immunofluorescence staining of the liver with the macrophage markers CD68 (red) and CD163 (green). (**d**) Periodic acid–Schiff staining of neonatal and 30 day livers from BBc and BBdp rats (n = 6–12), yellow arrows indicate extramedullary hematopoiesis (EMH) clusters. (**e**) Quantification of the number of EMH clusters/mm^2^. (**f**) EMH clusters and CD68^+^ cells. (**g**) Percentage of CD68^+^ cells per EMH cluster. (**h**) Comparison of number of EMH clusters in neonates, 30 day and 60 day BBc and BBdp rats. (For simplicity, data for each age are presented on the same axis). Data were analyzed using unpaired t-test with Welch’s correction (GraphPad 8) and are expressed as mean ± SD.

The majority of tissue-resident macrophages in the body, Kupffer cells, reside in the liver and play a role in immune regulation, antimicrobial defenses and glucose homeostasis. Because we observed immune changes in liver and macrophages are important players in pancreas of young BBdp rats^3,10^, we investigated the macrophage marker CD68^+^ in neonate and 30 day liver. There was a decrease in macrophage cells in neonates which persisted at 30 days (Fig. 1a,b). Co-staining with CD68 and CD163, a marker for M2 macrophages and Kupffer cells, confirmed that most macrophages in the liver display an anti-inflammatory tolerogenic phenotype under normal physiological conditions in control animals (Fig. 1c).

Extramedullary hematopoiesis (EMH) is a homeostatic process that develops during fetal maturation and decreases in infancy^11^. In some pathological conditions, EMH sites can reappear in different tissues to support hematopoiesis and formation of new immune cells. EMH clusters were decreased in neonatal BBdp compared with BBc rats (Fig. 1d,e). As BBc animals aged, we observed the expected decrease in the number of EMH clusters. However, the reduction observed in BBdp rats was modest, shifting from ~70% decrease in neonates to an increase approaching 40% and 70% at 30 and 60 days, respectively (Fig. 1e,h). Previous studies have shown an interdependence between liver resident macrophages and the development of EMH^12,13^. In BBdp neonatal liver there were fewer macrophages surrounding EMH clusters (Fig. 1f,g). Therefore, a key immune population associated with the stem cell niche for blood and immune cell populations in liver was disturbed in the neonatal period. Macrophages are associated with erythroblastic islands in bone marrow where proliferation and differentiation of erythrocyte precursors occurs^14^. This partnership can be impaired in various pathological conditions^15^. To our knowledge, this is the first report of fewer EMH clusters in neonatal liver of diabetes-prone rodents.

### Innate immune response genes are downregulated in the liver of prediabetic neonatal rats

**Figure 2:**
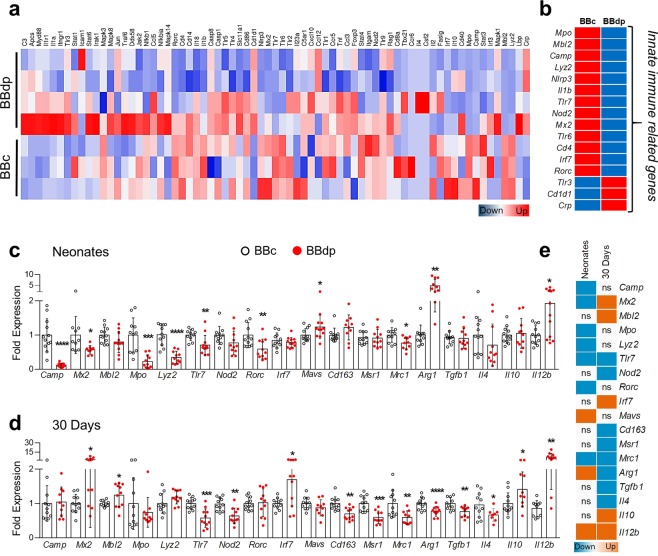
Innate immune gene expression in the liver of neonates and 30 day prediabetic rats. (**a**) Heatmap of Innate and Adaptive Immune Response PCR array gene expression from BBc and BBdp rat neonatal liver. (**b**) Summary of selected genes from the array showing significant differences (≥1.4 fold-change, p < 0.05) in BBdp rats. Validation of target genes was performed in liver samples (n = 10–12) from (**c**) neonates and (**d**) 30 day rats; BBc (open circle) and BBdp rats (red filled circles). (**e**) Summary of validated genes by RT-qPCR. Data were analyzed using unpaired t-test with Welch’s correction (GraphPad 8) and are expressed as mean ± SD. P values: * ≤ 0.05, ** < 0.01, *** < 0.001, **** < 0.0001.

To further characterize the very early immune events in the liver we performed a PCR immune array analysis of neonatal livers from BBc and BBdp rats (Fig. 2a). Most of the significant genes from the PCR array were downregulated in BBdp rats (Fig. 2b). Bioinformatic analysis using STRING^16^ showed enrichment of networks for biological processes associated with innate immunity involving antiviral and antimicrobial responses.

### Gene expression analysis of immune genes in BBdp rat liver

To confirm the results from the PCR immune array, we validated selected genes in neonate and 30 day BBdp and BBc liver by RT-qPCR. The antimicrobial genes *Camp, Mx2, Mpo, Lyz2* and *Tlr7* were downregulated at 8 days, whereas *Mavs*, an antiviral response gene, was upregulated in neonates (Fig. 2c). *Rorc* (RORγ), known for its role in Th17 cell differentiation^17^, was decreased in neonates (Fig. 2c). RORγ has also been shown to regulate gluconeogenesis in association with the hepatic circadian clock^18^. At 30 days, *Tlr7* and *Nod2*, both involved in pathogen recognition, were downregulated. However, *Mbl2*, a mannose-binding lectin complement activator, *Mx2* (a type 1 interferon responsive GTPase) and *Irf7*, a master regulator of interferon related genes, were upregulated at 30 days (Fig. 2d).

To further investigate the reduction in the CD68^+^ population (Fig. 1a) and the imbalance in innate immune genes, we analyzed the immune status of the liver in young BBdp rats, including several M1 and M2 macrophage-associated genes (Fig. 2). Expression of the M2 marker *Mrc1* (CD206) was decreased in neonatal BBdp rats. At the same time, *Il12b* and *Arg1* expression was increased. Although arginase-1 is considered a marker of M2 macrophages, hepatocytes are the primary source of *Arg1* expression in liver^19^. At 30 days, M2 macrophage markers *Cd163*, *Mrc1* and *Msr1* (CD204) were decreased in diabetes-prone animals (Fig. 2d). *Tgfb1* and *Il4* expression was decreased whereas expression of *Il10* was elevated in 30 day BBdp rats. *Il12b*, which can be expressed by activated macrophages remained upregulated at 30 days (Fig. 2d,e). We have previously shown that the proinflammatory (M1) cytokines *Il1b* and *Tnf* were increased in 30 day BBdp liver^3^. These results reveal impairment in neonates of genes involved in the innate immune response, which at 30 days appeared to have impacted the tolerogenic phenotype of the liver, favouring a gene signature characteristic of M1 macrophages.

### Metabolic genes associated with lipid homeostasis

**Figure 3:**
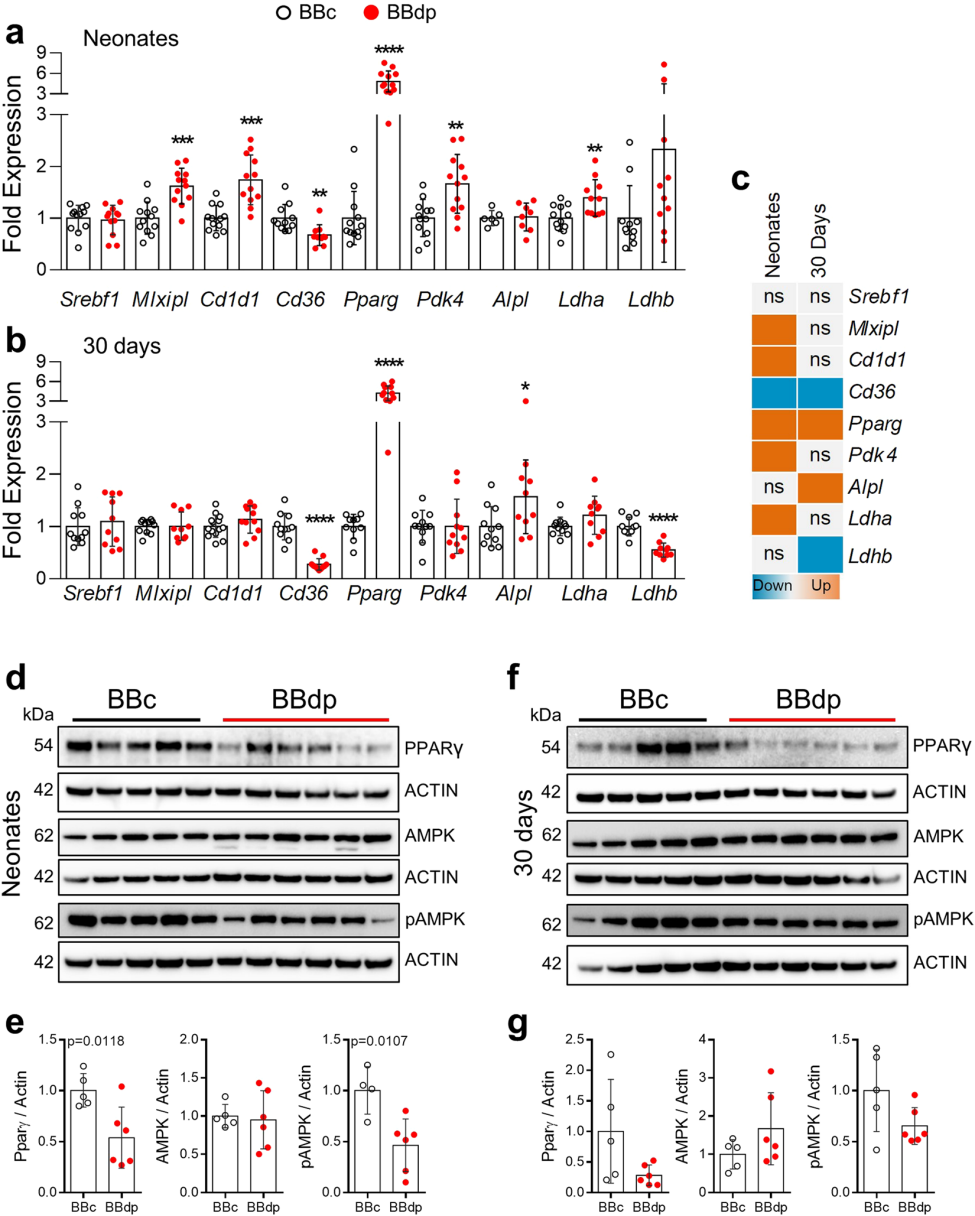
Metabolism related gene and protein expression in neonate and 30 day rat livers. Expression of glucose and lipid metabolism related genes was investigated in liver samples from (**a**) neonates and (**b**) 30 day BBc (black open circle) and BBdp rats (red filled circles) (n = 10–12). (**c**) Summary of genes analyzed using RT-qPCR. PPARγ, AMPKα, pAMPKα protein expression in neonate (**d**) and 30 day livers (**f**) (n = 5–6). Quantification was by densitometric analysis of chemiluminescence signal (**e**,**g**); proteins of interest were normalized to expression of β-actin on the same blot and individual animals were normalized to the mean value of the control animals (full length blots in Suppl Figs). Data were analyzed using unpaired t-test with Welch’s correction (GraphPad 8) and are expressed as mean ± SD.

One of the main functions of the liver is the control and balance of glucose and lipid metabolism. Apart from the immune signature we observed (Figs 1 and 2), our previous report^3^ also revealed a metabolic imbalance, particularly among genes associated with lipid metabolism such as *Gnpat* and *G0s2*^3^. Moreover, the PCR array analysis in Fig. 2 showed upregulation in BBdp rats of *Cd1d1*, which encodes a lipid antigen presentation molecule. Validation of *Cd1d1* confirmed this gene was upregulated in neonatal liver (Fig. 3a) and pancreas (Suppl. Fig. 1).

We investigated the expression of two of the main transcriptional regulators of lipogenic and glycolytic enzymes *Srebf1* and *Mlxipl* (Fig. 3a). *Srebf1* encodes the sterol regulatory element binding protein 1 (SREBP1) and *Mlxipl* encodes the carbohydrate response element-binding protein (ChREBP). *Srebf1* was not different, however, *Mlxipl* was upregulated in neonatal liver (and pancreas, Suppl. Fig. 1) as was *Pdk4* and *Pparg* (Fig. 3a), two downstream genes that respond to changes in glucose and lipid metabolism^20^. In contrast, fatty acid receptor *Cd36* was downregulated in both neonates and 30 day BBdp rats. *Alpl*, *Ldha and Ldhb* are genes that code for proteins routinely measured in the clinic to evaluate liver dysfunction. *Ldha* was upregulated in neonates. *Ldhb* was increased in neonates (p = 0.09) and downregulated at 30 days. *Alpl* was upregulated in 30 day animals.

*Pparg* was strongly increased in neonates and 30 day BBdp rats, however protein levels were significantly lower in neonates and remained low at 30 days (not statistically significant, Fig. 3d–g). Another key metabolic regulator of hepatic steatosis^21^ is the energy sensor 5′ adenosine monophosphate-activated protein kinase (AMPK). Phosphorylated AMPKα (pAMPKα) was initially lower in neonate liver but showed no difference at 30 days (Fig. 3d–g); total AMPKα protein levels were similar in BBc and BBdp rats.

### Evidence of steatosis and lipid dysregulation in the liver of young BBdp rats

**Figure 4:**
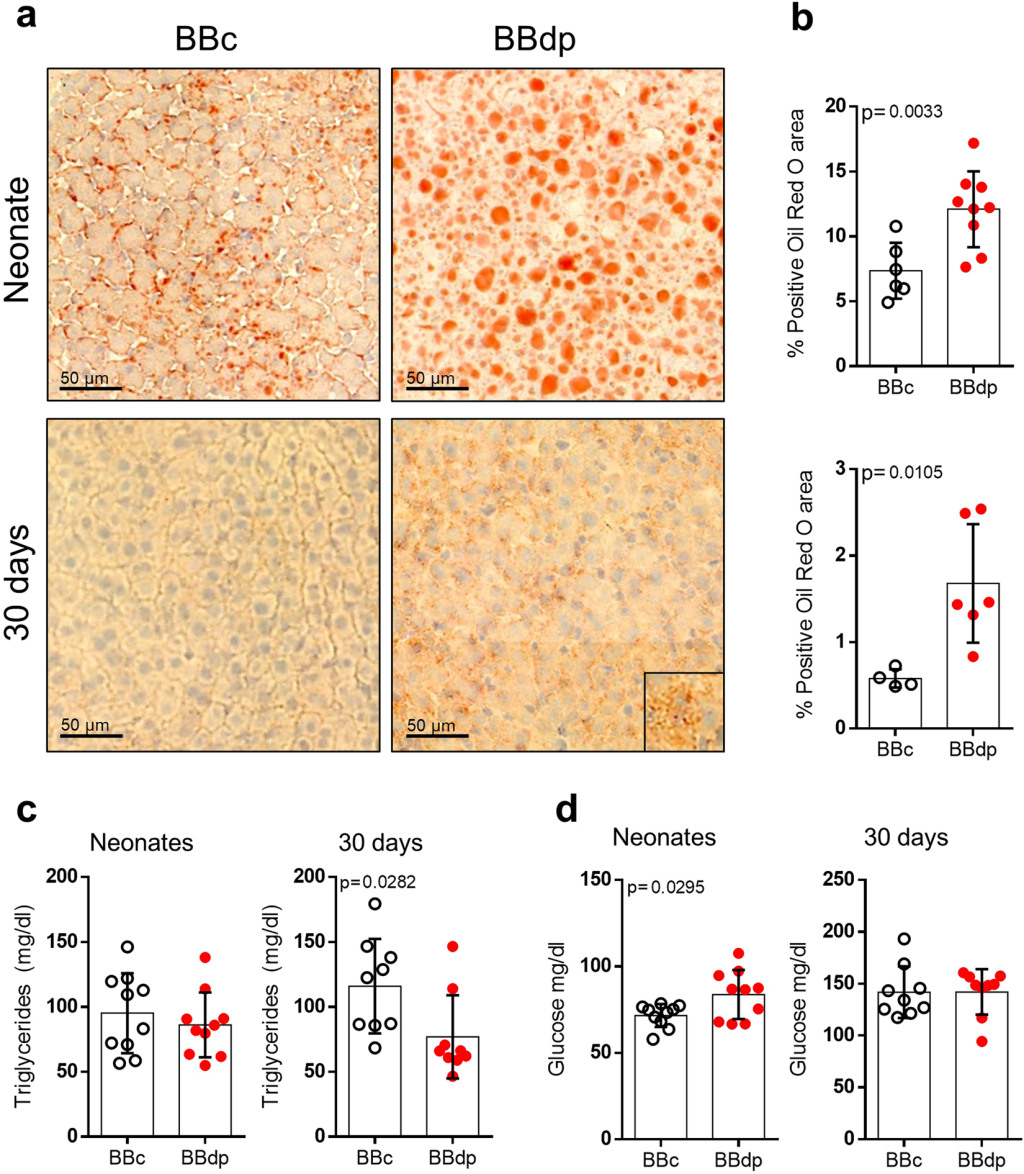
Prediabetic rats have higher lipid accumulation. (**a**) Neonatal and 30 day livers from BBc and BBdp rats were stained with Oil red O for detection of lipids (n = 4–9). Inset shows magnified area of lipid accumulation. (**b**) Percentage of Oil red O^+^ area in the livers. Serum levels (n = 10) of triglycerides (**c**) and glucose (**d**) in neonates and 30 day BBc (open circle) and BBdp rats (red filled circles). Data were analyzed using unpaired t-test with Welch’s correction (GraphPad 8) and are expressed as mean ± SD.

We next analyzed the lipid content in liver tissues by Oil red O staining. In BBdp neonate liver, there was a striking accumulation of lipid droplets (Fig. 4a,b). Although lipid accumulation was much lower at 30 days than in neonates, BBdp rats still displayed an increase compared with controls. Lipid accumulation can result in increased levels of triglycerides in the blood as well as impaired glucose uptake in liver, a process that can drive insulin resistance. Serum from neonates and day 30 rats was analyzed for triglycerides and glucose. In neonates, no difference was observed for triglycerides (Fig. 4c). Serum glucose was higher in neonatal BBdp compared with BBc rats, though still within normal glycemic range (Fig. 4d); values at 30 days did not differ. Triglycerides were down at 30 days.

### Evidence of steatosis and decreased macrophage population in the liver of NOD mice

**Figure 5:**
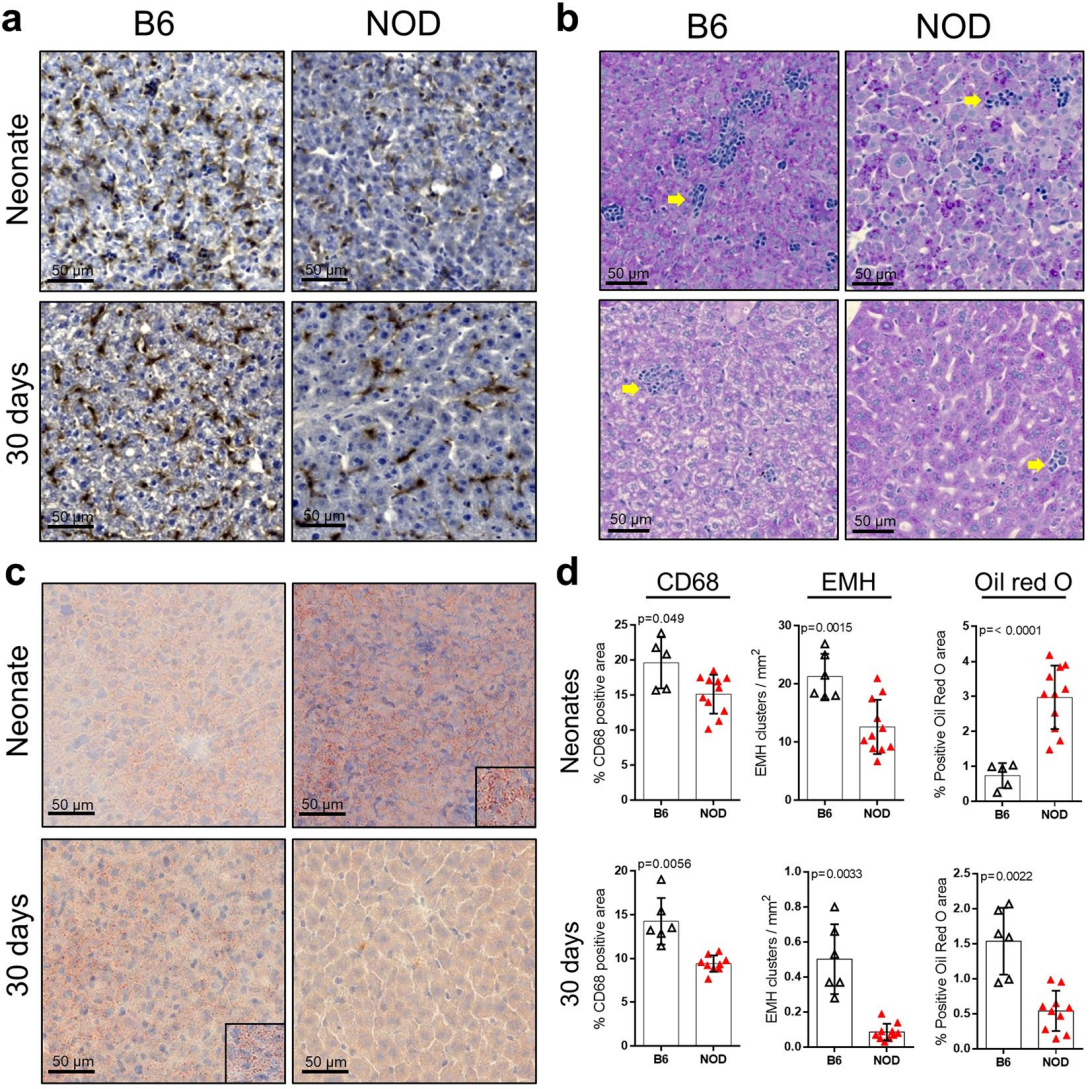
Reduced macrophages, EMH clusters and higher lipid accumulation in the livers of NOD mice. Livers from neonatal and 30 day C57BL/6J and NOD/ShiLtJ mice were labelled using IHC for the macrophage marker, CD68 (**a**), with Periodic acid–Schiff (**b**) and with Oil red O for detection of lipids (**c**). Inset shows magnified areas of lipid accumulation. (**d**) Quantification of CD68^+^ area, EMH clusters/mm^2^ and percentage of Oil red O^+^ area in B6 (open triangle) and NOD mice (red filled triangles). Data (n = 5–12) were analyzed using unpaired t-test with Welch’s correction (GraphPad 8) and are expressed as mean ± SD.

Considering the disruption of metabolism, ER stress and innate immunity that we observed in BBdp rats, we quantified the number of macrophages, EMH clusters and lipid load in NOD liver. CD68^+^ area was diminished in neonates and 30 day NOD mice (Fig. 5a,d). Although there were many more EMH clusters in neonatal control mice than in control rats, both diabetes-prone rodents showed fewer clusters than in controls (Figs 1h and 5d). However, at 30 days, NOD mice displayed a striking reduction in EMH clusters compared with control mice (Fig. 5b,d) whereas the number of clusters in BBdp rats was increased compared with BBc (Fig. 1h). Oil red O staining confirmed that lipid metabolism was disturbed in the NOD neonate liver (Fig. 5c,d).

### Changes in ER stress, lipid metabolism and immune genes also occur in NOD mice

We evaluated selected metabolic and immune genes in the pancreas and liver of NOD and control C57BL/6J mice. In contrast to neonatal BBdp rats^3^, which showed upregulation of several UPR genes, only *Ern1* (IRE1α) was increased in neonatal NOD mouse pancreas (Suppl. Fig. 2). At 30 days, all three branches of the UPR (*Ern1, Eif2ak3* (PERK) *and Atf6*) were upregulated. While there was no change in neonates, 30 day NOD mice displayed increased *Ins1, Ins2, Gcg* and *Srebf1* expression (Suppl. Fig. 2). Unlike BBdp rats, where *Ins1* was downregulated in neonates, both *Ins1* and *Ins2* were down at 30 days and glucagon was upregulated at both ages^3^. Thus, both BBdp and NOD have different pancreatic gene signatures than controls. However, in the case of NOD, the differences we observe could also be attributable to strain differences with C57BL/6.

**Figure 6:**
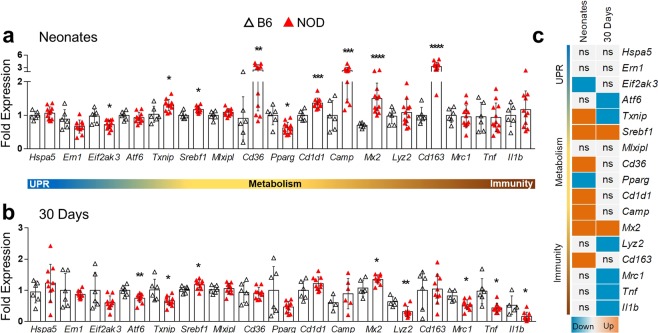
Gene signature in neonatal and 30 day liver of NOD mice. Expression of genes related to ER stress, metabolism and immunity in neonates (**a**) or 30 day (**b**) NOD mice. (**c**) Summary of validated genes by RT-qPCR. Data (n = 4–12) were analyzed using unpaired t-test with Welch’s correction (GraphPad 8) and are expressed as mean ± SD.

Compared with BBdp rat liver where we observed widespread upregulation of the UPR machinery in neonates followed by inhibition at 30 days^3^, NOD mouse liver displayed only minor changes in UPR gene expression. In neonatal NOD mouse livers (Fig. 6a), the chaperone *Hspa5* was unchanged and only one branch of the UPR was downregulated, *Eif2ak3*. By 30 days, only *Atf6* was downregulated (Fig. 6b). *Txnip*, which is involved in the UPR and metabolism, was up in neonates and down by 30 days in both BBdp rats and NOD mice.

Lipid related genes *Srebf1* and *Cd36* were upregulated in neonatal NOD mice, whereas *Pparg* was down. Also, in the neonatal period, the innate immune genes *Camp, Mx2, Cd1d1* and *Cd163* were strongly upregulated in liver (Fig. 6a). With the exception of *Srebf1* (lipid metabolism) and *Mx2* (antiviral) which remained up, most of the early upregulated genes returned to control levels by 30 days (Fig. 6b). However, in contrast to BBdp rats, the proinflammatory genes *Tnf* and *Il1b* were downregulated by 30 days.

These results showed that genes involved in ER stress, metabolism and immune response are dysregulated in both models. The later appearance of these dysregulated processes in NOD compared with BBdp may be related to the late appearance of the disease in this model. Interestingly, both animal models showed signs of metabolic imbalance and hepatic steatosis very early in life. The fatty liver phenotype may be a key focus for future research, as this pathological condition can cause or aggravate other ongoing pathologies such as T2D.

## Discussion

The liver is the command centre for function and control of metabolism. As such it must maintain close communication with several key organ systems that assist in this process, namely the gastrointestinal tract, pancreas, neuronal network and immune system. β-cells act as a rheostat that manages glucose levels by sensing various stimuli from the gastrointestinal tract and liver to release just the correct amount of insulin^22^. In turn, insulin and glucose control the metabolic activities of the liver, transcriptionally regulating lipogenesis, gluconeogenesis and glycogen storage. Additionally, the liver constantly filters the blood and receives large quantities of microbial and dietary molecules from the gut, particularly in the neonatal period. Despite this, the liver is mainly a tolerogenic immune organ that represents an intersection point between metabolism and immunity that is key to maintaining physiological balance. When this balance is impaired, inflammation can result.

Kupffer cells are the most abundant macrophage population in the body and are key players in host defense, metabolic regulation and immune tolerance in the liver^23^. In the present study, we demonstrate that young BBdp rats and NOD mice have fewer CD68^+^ macrophages than control animals (Figs. 1a,b and 5a,d), associated with increased expression of proinflammatory cytokine genes in neonates and decreased expression of M2 markers in 30 day BBdp rats (Fig. 2d,e). We speculate that the Kupffer cells of diabetes-prone rats have a proinflammatory, M1 macrophage-like phenotype. M1 macrophages have been implicated in the development of both type 1 and type 2 diabetes^24^ and in non-alcoholic fatty liver disease (NAFLD)^25^.

Macrophage cells are the first to be recruited to the islet in the development of T1D^10^. In addition, polarization of adipose-resident macrophages to an M1 phenotype contributes to the development of insulin resistance and T2D^26^. Furthermore, suppressing the pro-inflammatory factor NF-κB in Kupffer cells restored insulin sensitivity^27^. While expression of the M2 marker *Mrc1* was decreased at 30 days, our current study did not show a definitive M1 signature in the liver of neonatal and 30 day NOD mice. However, previous studies have shown that T1D can be prevented in NOD mice by the adoptive transfer of M2 macrophage cells^28^, suggesting that M1/M2 balance is altered in these animals. Macrophages have been shown to have an interdependent role in the formation of EMH clusters^12^. Neonatal livers from BBdp rats and NOD mice showed a marked reduction in both CD68^+^ cells and EMH clusters, however over time EMH clusters increased in rats, a process observed in pathological conditions and linked to inflammation^11^. Because macrophage populations did not increase, this could suggest that other immune populations are formed at those sites in response to the surrounding pro-inflammatory environment. Further investigation of hepatic M1/M2 balance throughout diabetes pathogenesis in the NOD model is needed to clarify these findings.

Both BBdp rats and NOD mice had dysregulated expression of several genes associated with antimicrobial and innate immune responses. Cathelicidin antimicrobial peptide (CAMP) is associated with anti-inflammatory M2 macrophages and is decreased in the gut and pancreas of BBdp rats^29,30^. We show a reduction of antimicrobial and M2 macrophage-associated markers and an increase in proinflammatory genes in BBdp liver (Fig. 2b,c). This suggests that antimicrobial defenses are impaired in BBdp rats and could contribute to the intestinal dysbiosis we previously observed^30^. CAMP has been shown to downregulate CD36 and inhibit the formation of liver steatosis^31^, consistent with the decreased *Camp*, *Cd36* and increased lipid content we observe in neonates. In contrast, neonatal NOD mice showed increased gene expression of *Camp* and *Mx2* and decreased or unchanged expression of inflammatory cytokines. Neonatal NOD mice have altered gut microbiota compared with C57BL/6 mice, accompanied by increased numbers of hepatic granulocytes^32^. This could partially explain the increase in antimicrobial genes (Fig. 6).

It is important to acknowledge that allelic variations at loci not linked to T1D could be responsible for the phenotypic differences we observe. Apart from the *Ian5* locus, which imparts increased risk for T1D in BB rats, the extent of genetic polymorphism between BBDP and control, BBDR rats is ~15% across the rest of the genome. Therefore, it is possible that other non-T1D-related loci control the phenotypes we observed. Similarly, in the absence of extensive genetic analyses that are beyond the scope of the current study, it is not possible to rule out the role of genetic loci unlinked to T1D in the phenotypic differences observed between C57BL/6J and NOD mice. The analysis of NOD-related strains such as NOR mice would not allow to rule out the role of non-*Idd* loci since these two strains exhibit ~20% genetic polymorphism across the whole genome.

Accumulation of lipids in the liver is the hallmark of NALFD and is sometimes accompanied by inflammation and fibrosis. NALFD has been identified as a predictive factor for the development of T2D^33^. The association of T1D with NALFD is more controversial, with some studies finding no^34^ or a minor^35^ association with NAFLD while other studies report increased NALFD in patients with T1D^36,37^. Importantly, none of these studies examined very young, prediabetic individuals. There are reports that dysregulated lipid metabolism occurs before islet autoantibodies appear^38^. Indeed, metabolic dysregulation may even be apparent in cord blood of T1D patients^8^.

Our study identified liver steatosis in very young animals long before disease onset. This appears to be a transient phenotype that manifests in infancy during high fat feeding and is reduced after weaning to a more complex, mainly carbohydrate and mixed protein diet (Figs 4a and 5c). This was associated with decreased PPARγ and pAMPKα in neonatal BBdp liver. AMPK acts as a metabolic switch, regulating ATP levels in response to cellular and systemic stress^39^ and inhibits lipogenesis through regulation of SREBP-1c^40^, ChREBP^41^ and PPARγ^42^. Commonly known for its role in adipogenesis^43^, PPARγ has been shown to be upregulated in steatotic livers of both humans^44^ and rodents^45^. Conversely, increased *Pparg* expression was associated with improved metabolic control in obese rats after 2 months of increased aerobic activity and vitamin D supplementation^46^ and PPARγ agonists may also reverse steatohepatitis (NASH)^47^. Thus, it is difficult to elucidate whether PPARγ has a protective or detrimental role in liver steatosis. Importantly, PPARγ is also a regulator of M2 polarization^43^ and the decrease in PPARγ protein observed in neonatal BBdp rats is consistent with a loss of tolerance in the BBdp liver (Fig. 3d,e). Interestingly, we also observed an increase in *Cd1d1* expression in the liver of neonatal BBdp rats (Figs 2a,b and 3a,c) and NOD mice (Fig. 6a,c) and also in the pancreas of neonatal and 30 d BBdp rats (Suppl. Fig. 1A,C). CD1D is an MHC-like molecule that presents lipid antigens such as glycerophospholipids and sphingolipids to NKT cells^48^. Abnormal sphingolipid metabolism has recently been described in patients with T1D and NOD mice^49^. The increased *Cd1d1* expression together with other innate immune genes, suggests that recognition of antigens of lipid origin may be an early target of the inflammatory immune response.

Our previous study suggested that BBdp neonates display an insulin resistant profile^3^ consistent with a marked accumulation of lipid in hepatocytes (Fig. 4). Serum triglycerides did not differ in neonates and even declined at 30 days when lipids were still higher in BBdp hepatocytes. Thus, lipids are being retained in the liver and not being secreted into the circulation. The cause of this difference is unclear but could indicate a reduced capacity to handle the usual high fat diet that neonates receive compared with the low fat, high carbohydrate diet fed at weaning. An additional exacerbating effect could involve defects in Ca^2+^ homeostasis in BBdp rats^50^ which could impact mitochondrial capacity for fatty acid oxidation^51^, ER capacity to manage cellular stress^52^, and lysosomal participation in lipid synthesis and turnover^53^. This could also explain why lipid accumulation is more striking in neonatal BBdp rats compared with NOD mice. Nonetheless, it is remarkable that both models share many similarities in lipid, UPR and immune imbalance, despite differences in pathogenesis. Our findings suggest that liver steatosis induced by disrupted insulin signaling may be an early predictor of T1D development and this could result in a loss of immune tolerance in the liver.

Both BBdp rats and NOD mice have been shown to have disrupted insulin levels early in life. Previous reports have demonstrated that NOD mice have increased levels of insulin^54,55^. This is consistent with our data (Suppl. Fig. 2) and with the increased hepatic *Srebf1* expression observed in neonatal NOD mice. SREBP proteins are key activators of lipogenesis in the liver and are activated by increased insulin levels. Conversely, we observed decreased insulin in the BBdp pancreas^3^ and a small but significant increase in blood glucose levels (Fig. 4d). This was accompanied by an increase in *Txnip*^3^ and *Mlxipl* expression in the liver, suggesting that this increase in blood glucose was physiologically relevant. *Mlxipl* encodes ChREBP, also a master activator of lipogenesis that is induced by glucose.

It is just beginning to be recognized that inter-organ communication could play a role in diabetes pathogenesis^9,22^. It has been recognized in the pathogenesis of T2D^9^ and changes in the liver are known to occur in overt T1D^56^, but the role of inter-organ communication in the early developmental stages of T1D remains an open question. Crosstalk between the liver and the immune system has the potential for integrating the sensing of nutrients and pathogens^57^, and could begin to explain the effects of diet and gut microbiota on T1D development. Recent advances in non-invasive imaging methods such as magnetic resonance spectroscopy and ultrasound elastography may enable testing of this hypothesis in children at-risk of developing T1D^58,59^.

## Methods

### Animals

Diabetes-prone BioBreeding (BBdp) and control BioBreeding (BBc) rats were maintained as previously described^3^. BBdp dams were non-diabetic and were mated with diabetic males. Young rats were weaned between 21 and 23 days and neonates were housed with their dams. We randomly sampled neonatal rats from each litter. At 30 days, we used equal numbers of males and females. Liver samples were harvested at 8 or 30 days and frozen in liquid N_2_ or fixed in Bouin’s fixative and stored in 70% ethanol. Pancreas samples were homogenized in RNA extraction buffer before freezing. All procedures related to animal care, maintenance and tissue collection were approved by the University of Ottawa Animal Care Committee. All experiments were performed in accordance with relevant guidelines and regulations.

NOD/ShiLtJ (NOD) and C57BL/6J mice (Jackson Laboratories, Bar Harbor, MA, USA) were maintained at the Sunnybrook Research Institute (Toronto, ON, Canada). NOD dams were screened 2–3 times/week and none was diabetic during gestation and nursing. Median diabetes incidence was not significantly different in male (~65%) and female (~75%) NOD mice by 126 days. Liver and pancreas tissue were taken at necropsy from 10 day or 30 day NOD mice (n = 12, 6 males and 6 females) or age- matched C57BL/6J mice (n = 6, 3 males, 3 females). Tissues were frozen in liquid N_2_ or fixed in formalin and stored in 70% ethanol. All procedures were approved by the Sunnybrook Research Institute Animal Care Committee.

### Histology and Immunohistochemistry

Fixed tissues were embedded in paraffin, cut into 5 μm sections, and stained with hematoxylin-eosin, periodic acid–Schiff (University of Ottawa Histology Core Facility, Ottawa, ON, Canada) or used for immunohistochemistry. Following antigen retrieval (0.05% trypsin and 0.1% CaCl_2_ solution, pH = 7.8 for 20 minutes at 37 °C) rat liver sections were incubated with anti-rat CD68 (BioRad Laboratories, Hercules, CA, USA) and ImmPRESS® Polymer Reagents secondary reagent (Vector Laboratories Burlingame, CA, USA). Sections were then incubated with 3, 3-diaminobenzidine (DAB) and counterstained with hematoxylin. For immunofluorescence, the samples were thawed for 10 min at 4 °C, fixed in 4% paraformaldehyde for 30 minutes at room temperature, permeabilized for 20 minutes with 0.2% Triton X-100, and blocked for 45 minutes with Protein Block (Agilent, Santa Clara, CA, USA) before incubating with primary anti-CD68 and CD163 overnight at 4 °C. Alexa 488- or 555-conjugated secondary antibodies were used. Mouse CD68^+^ cells were detected in frozen tissues with anti-CD68 antibodies (BioRad Laboratories) in combination with DAB staining using ImmPRESS® Polymer Reagents. Oil Red O staining was performed using frozen tissues (University of Ottawa Histology Core Facility). For image acquisition for immunohistochemistry, we used Aperio Scanscope and Zeiss Axio Scan.Z1; additional analyses used Scanscope software and Fiji (imageJ). Immunofluorescence images were acquired using a Zeiss LSM 800/AxioObserver Z1 microscope.

### Immune array and qPCR validation

Hepatic immune gene expression was quantified in neonate BBc (n = 3) and BBdp (n = 4) rats using the RT² Profiler™ PCR Array Rat Innate and Adaptive Immune Response kit (Qiagen, Toronto, ON, Canada). Data are presented as a heatmap^60^. Additional analyses by RT-qPCR of target genes (≥1.4 fold-change, *p* < 0.05) were performed using liver and pancreas tissues from 8 and 30 day BBc and BBdp rats (n = 10–14) as described previously^3^. Selected genes were assessed in 10 and 30 day NOD (n = 10–12) and C57BL/6J mice (n = 6). RNA was extracted using the Nucleospin RNA extraction kit (Macherey-Nagel, Bethlehem, PA, USA) and reverse-transcribed to cDNA. Gene expression was quantified with TaqMan^TM^ probes (ThermoFisher Scientific and Integrated DNA Technologies, Coralville, IA, USA) on an Applied Biosystems^®^ 7500 Real-Time PCR System (ThermoFisher Scientific) using *Actb* as an endogenous control. Data are presented as fold-change (2^−ΔΔCt^) and were normalized to the mean value of age-matched control BBc rats or C57BL/6J mice.

### Metabolite assays

Glucose and triglyceride levels were measured in serum according to manufacturer’s instructions using Glucose Colorimetric Assay or Triglyceride Colorimetric Assay Kits (Cayman Chemical, Ann Arbor, MI, USA).

### Immunoblotting

Immunoblotting was performed as previously described^3^. Liver protein was extracted using RIPA buffer with protease and phosphatase inhibitors (ThermoFisher Scientific) and repeated freeze-thawing. 50 μg of total liver protein from 8 and 30 day BBc (n = 5) or BBdp (n = 6) rats was resolved on a 4–12% acrylamide gel (ThermoFisher Scientific) and transferred to PVDF membranes (BioRad Laboratories). Membranes were blocked with 5% skim milk or 5% BSA in TBS-Tween and probed with anti-PPARγ antibody (Santa Cruz Biotechnology, Dallas, TX, USA), anti-AMPKα, or anti-pAMPKα (Cell Signaling Technology, Danvers, MA, USA) overnight at 4 °C or with anti-β-actin-HRP (Cell Signaling Technology) for 1 hour at room temperature. Proteins were detected by chemiluminescence after incubation with HRP-conjugated secondary reagents using a BioRad Chemidoc imaging system. Densitometric analysis was performed using ImageLab software (BioRad Laboratories). Band intensity of proteins of interest was normalized to β-Actin expression on the same membrane.

### Statistical analyses and data availability

RT^2^ Profiler™ PCR array data were analyzed using the online GeneGlobe Data Analysis Centre (Qiagen). RT-qPCR, immunohistochemistry, histology, immunoblotting and metabolite data were analyzed using Student’s t-test with Welch’s correction (GraphPad Prism 8.0, San Diego, CA, USA). The datasets generated during and/or analyzed during the current study are available from the corresponding author upon reasonable request.

### Prior presentation

Part of this study was presented in abstract form at the 16th Immunology of Diabetes Society Meeting, London, U.K., October, 2018.

## Supplementary information

Supplementary Information
