# Supplementary material for: Dysregulated liver lipid metabolism and innate immunity associated with hepatic steatosis in neonatal BBdp rats and NOD mice
Source: Sci Rep. 2019 Oct 10;9:14594. doi: 10.1038/s41598-019-51143-7 (PMC6787248; doi:10.1038/s41598-019-51143-7)
Supplement: Supplementary file 1 — Supplementary Information [file 41598_2019_51143_MOESM1_ESM.pdf]

SUPPLEMENTARY INFORMATION

Dysregulated liver lipid metabolism and innate immunity associated with hepatic steatosis in neonatal BBdp rats and NOD mice

Serrano D[1], Crookshank JA[1], Morgan BS[1], Mueller RW[2], Paré M-F[1], Marandi L[3], Poussier P[3], Scott FW[1,4,5*]

[1]Chronic Disease Program, Ottawa Hospital Research Institute, Ottawa, Ontario, Canada

[2]Department of Pathology and Laboratory Medicine, Faculty of Medicine, University of Ottawa, Ottawa, Ontario, Canada

[3]Sunnybrook Research Institute, Toronto, Ontario, Canada

[4]Department of Medicine, University of Ottawa, Ottawa, Ontario, Canada

[5]Department of Biochemistry, Microbiology and Immunology, University of Ottawa, Ottawa, Ontario, Canada

Supplemental Fig.1. Serrano et al.

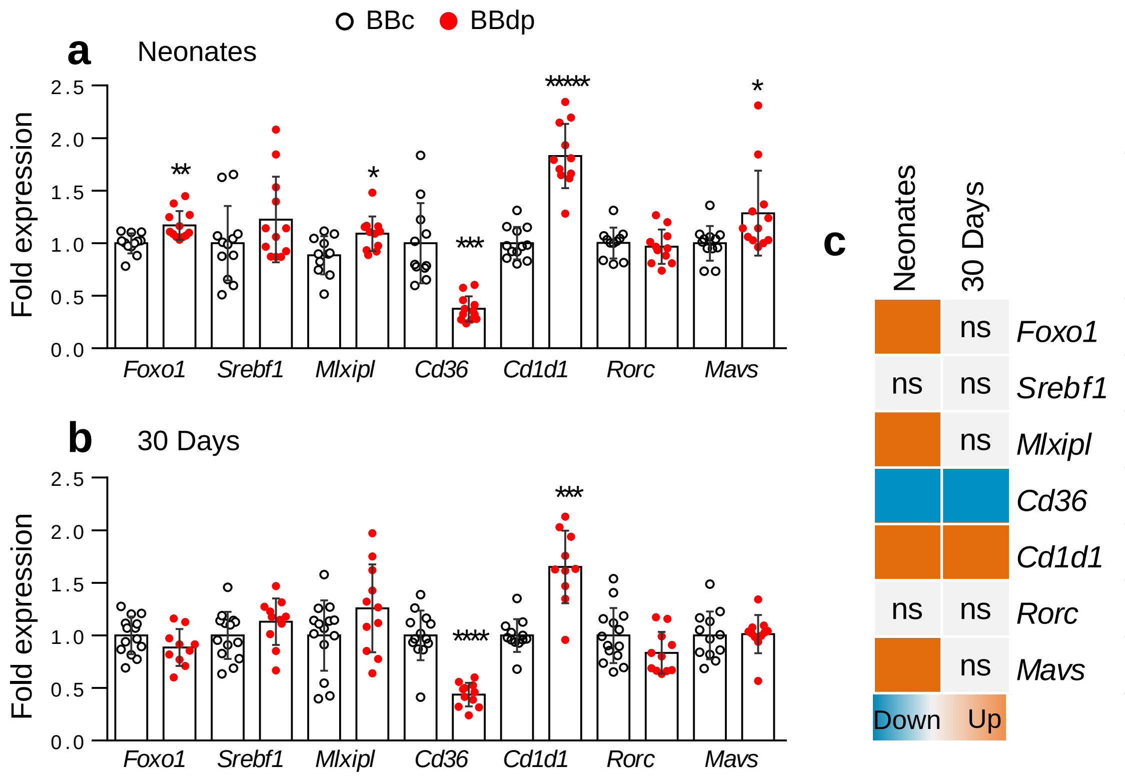

Supplemental Fig. 1. Metabolic imbalance in the pancreas of prediabetic BBdp rats. Expression of glucose and lipid metabolism related genes was investigated in pancreas samples from (A) neonates and (B) 30 day BBc (open black circles) and BBdp rats (red filled circles). (C) Summary of genes analyzed using RT-qPCR. Data (n=10-12) were analyzed using unpaired t-test with Welch's correction (GraphPad 68) and are expressed as mean ± SD. P-value: * ≤ 0.05, ** < 0.01, *** < 0.001, **** <0.0001.

Supplemental Fig.2. Serrano et al.

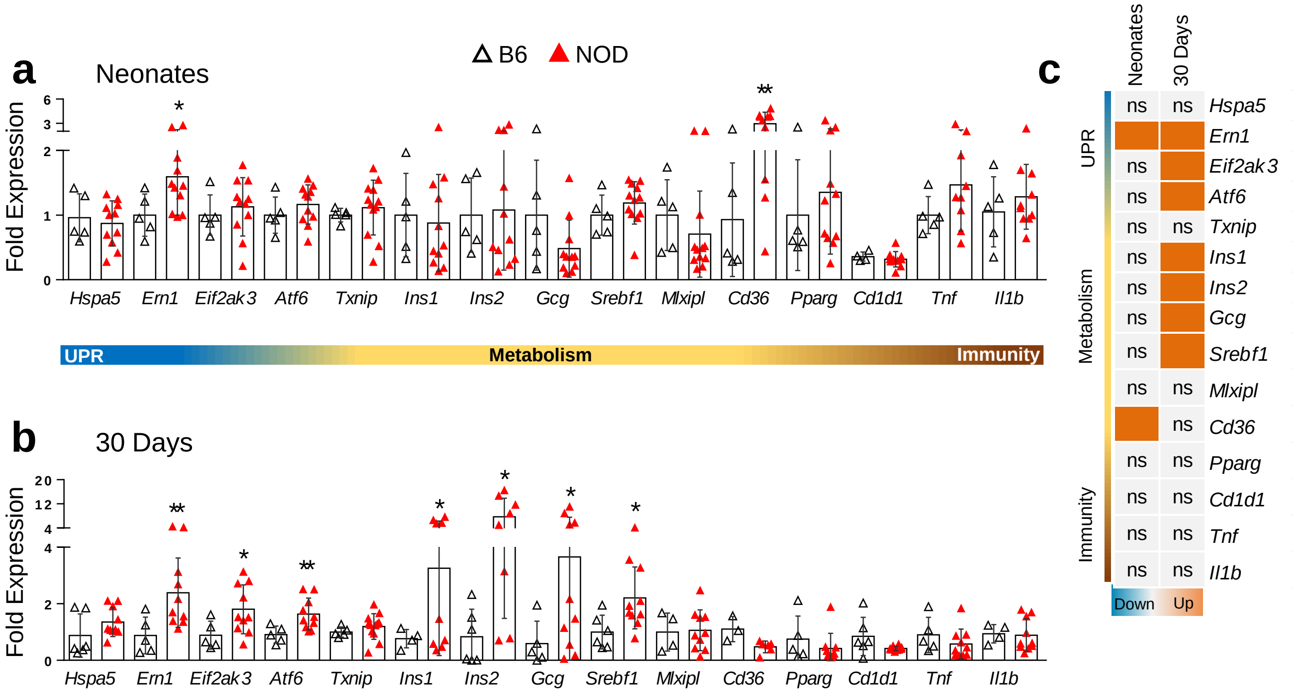

Supplemental Fig. 2. Gene signature in neonatal and 30 day pancreas of NOD mice. Expression of genes related to ER stress, metabolism and immunity in neonates (A) or 30 day (B) NOD (filled red triangles) and C57BL/6J (black open triangles) mice. (C) Summary of genes analyzed using RT-qPCR. Data (n=3-12) were analyzed using unpaired t-test with Welch’s correction (GraphPad 8) and are expressed as mean ± SD.

Supplementary information for western blots in **Figures 3 d,f**. Images show the raw data in the BioRad ImageLab analysis software before cropping for publication purposes.

A

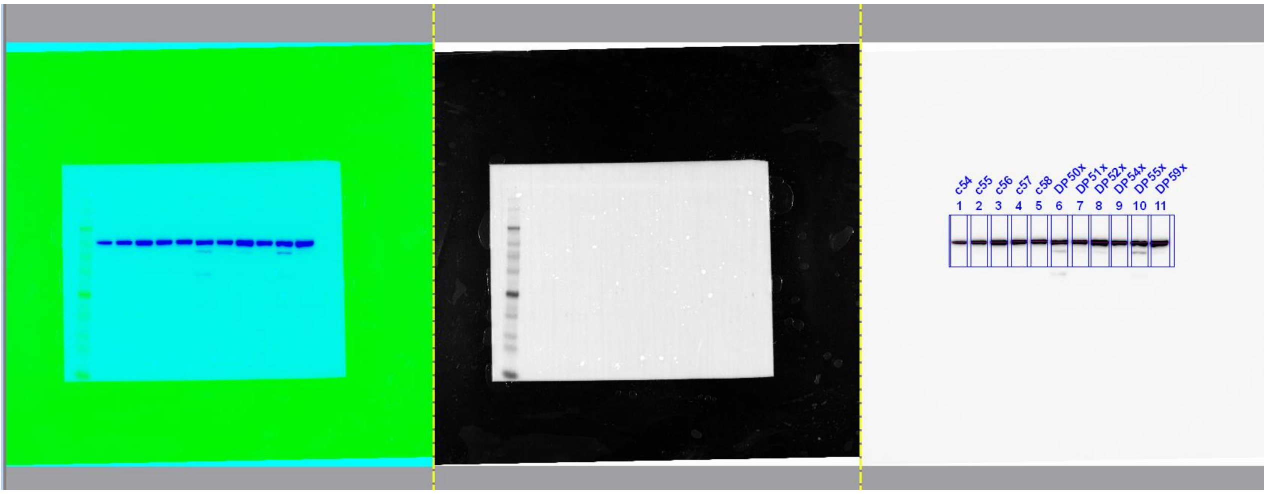

B

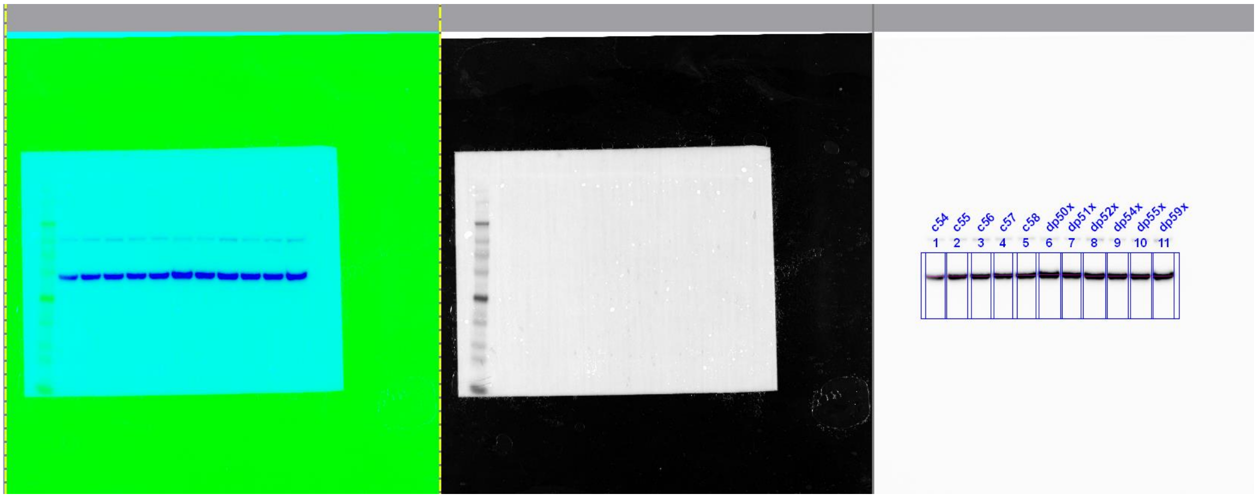

AMPK protein expression in neonatal rat liver Bands for AMPK were cropped around 62 kDa according to protein ladder **(a)** and the same membrane was later exposed to actin antibody and cropped around 42 kDa **(b)** Densitometry analysis was performed with the raw data using Image Lab 5.2 software from BioRad

A

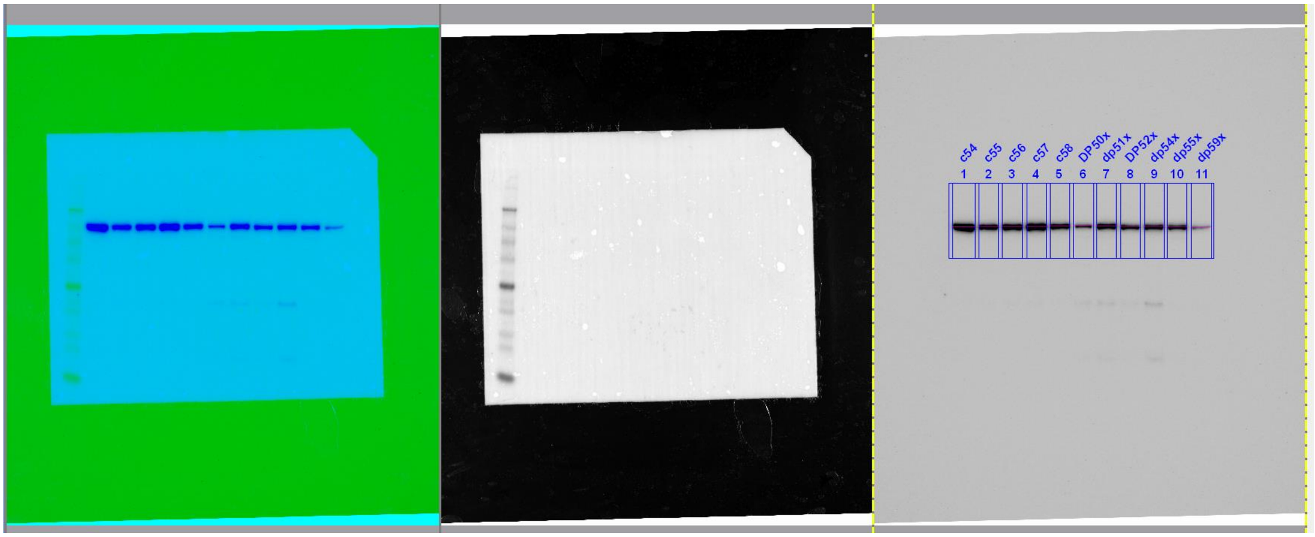

B

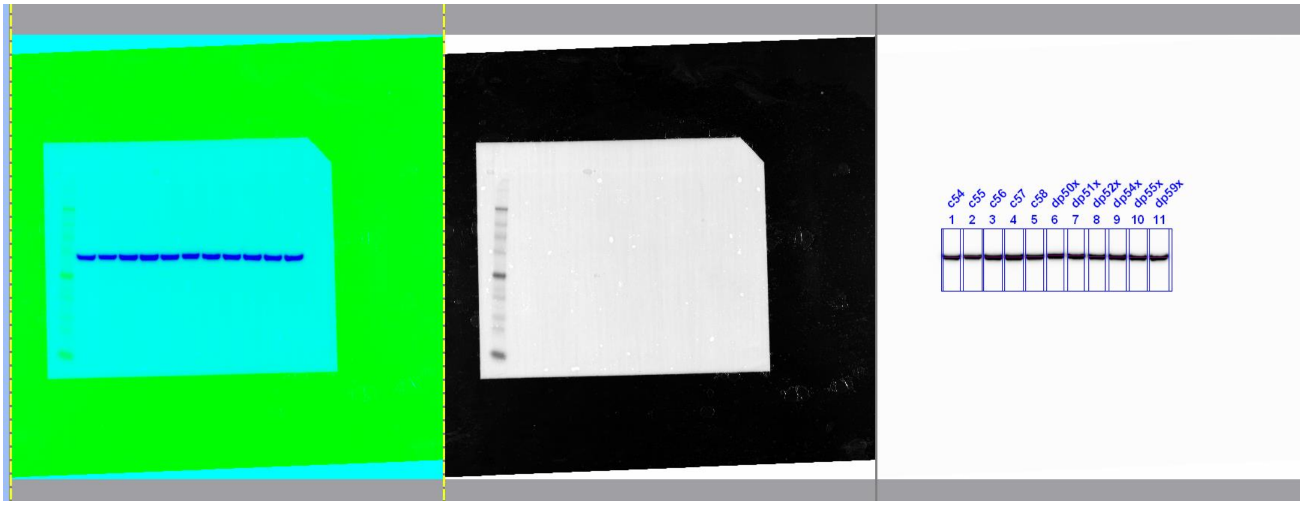

pAMPK protein expression in neonatal rat liver Bands for pAMPK were cropped around 62 kDa according to the protein ladder **(a)** and the same membrane was later exposed to actin antibody and cropped around 42 kDa **(b)** Densitometry analysis was performed with the raw data using Image Lab 5.2 software from BioRad

A

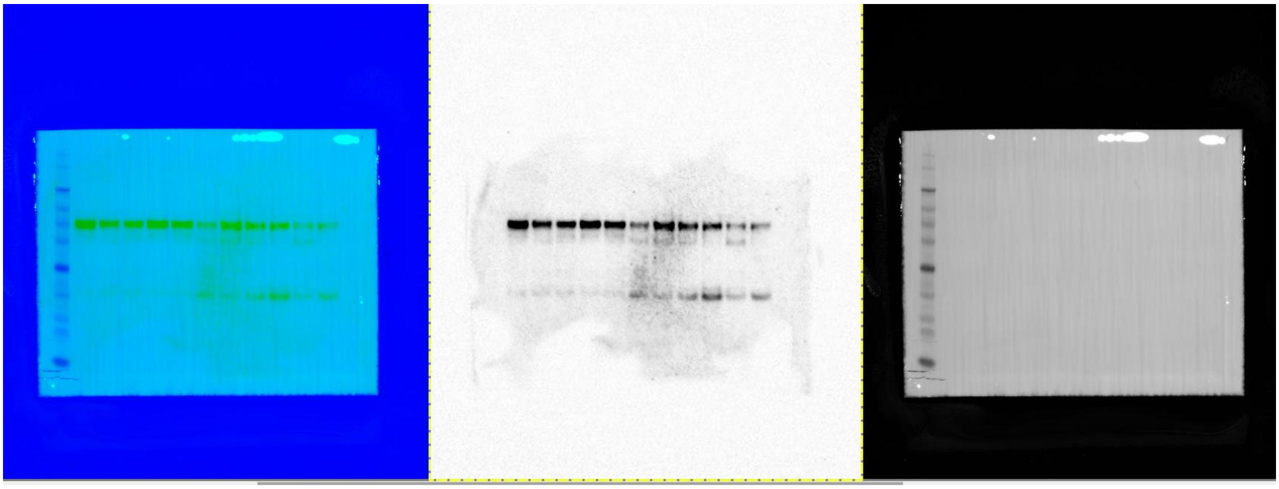

B

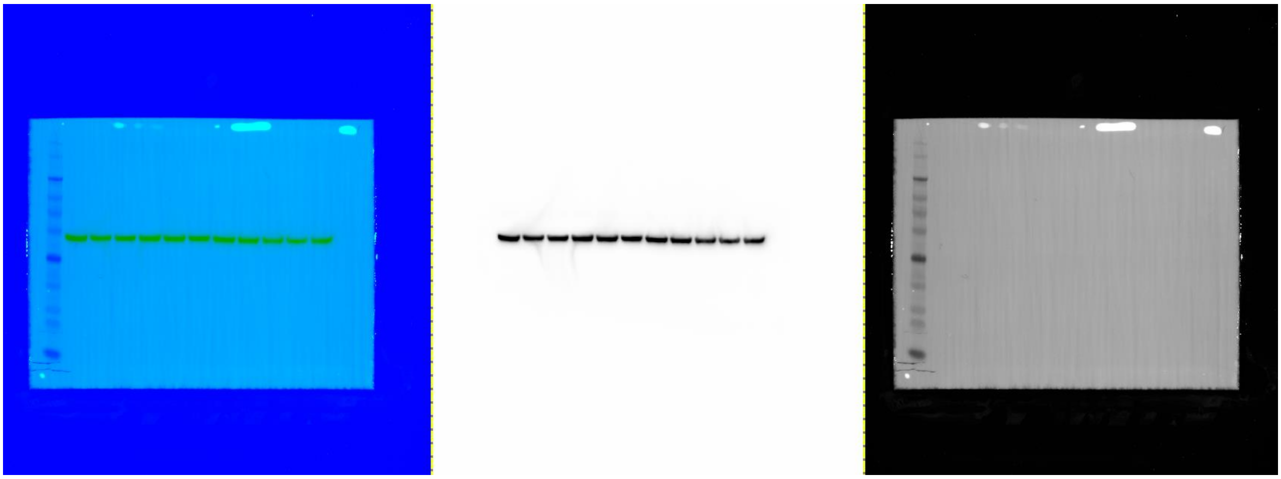

PPARɣ protein expression in neonatal rat liver Bands for PPARɣ were cropped around 54 kDa according to the protein ladder **(a)** and the same membrane was later exposed to actin antibody and cropped around 42 kDa **(b)** Densitometry analysis was performed with the raw data using Image Lab 5.2 software from BioRad

A

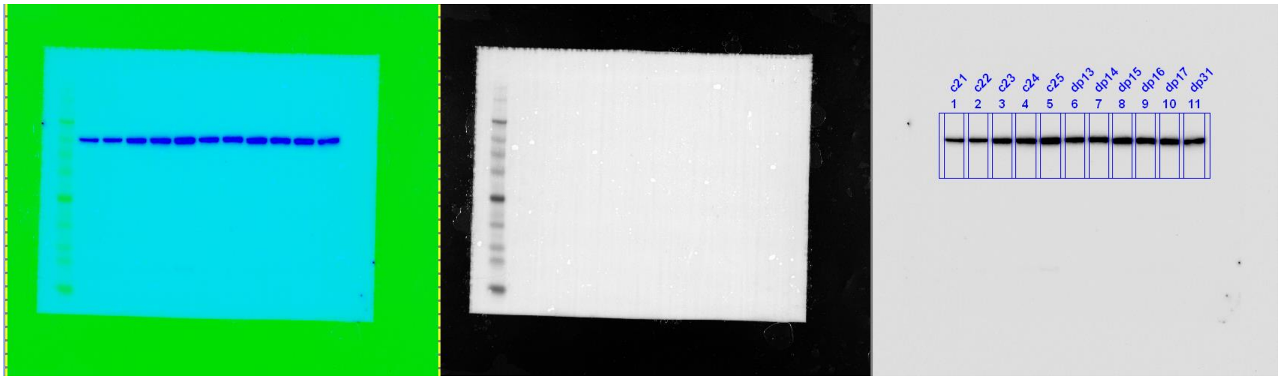

B

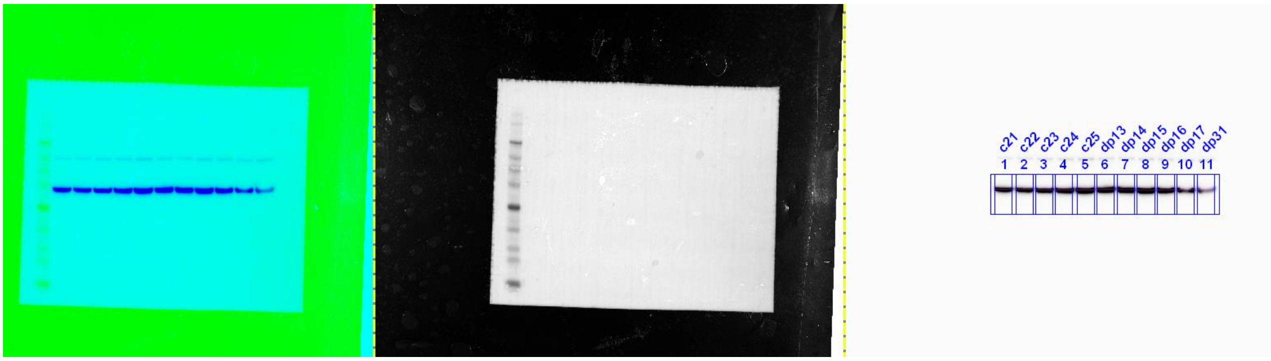

AMPK protein expression in 30 day rat liver Bands for AMPK were cropped around 62 kDa according to protein ladder **(a)** and the same membrane was later exposed to actin antibody and cropped around 42 kDa **(b)** Densitometry analysis was performed with the raw data using Image Lab 5.2 software from BioRad

A

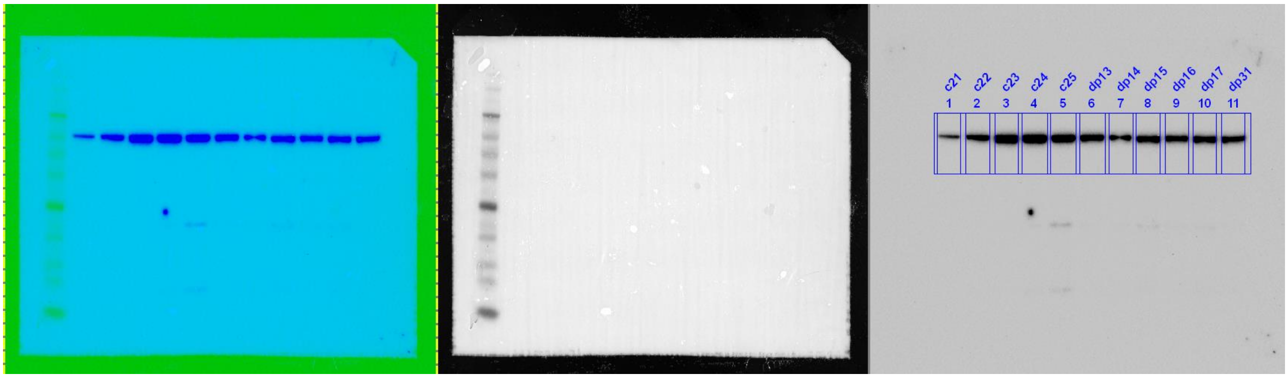

B

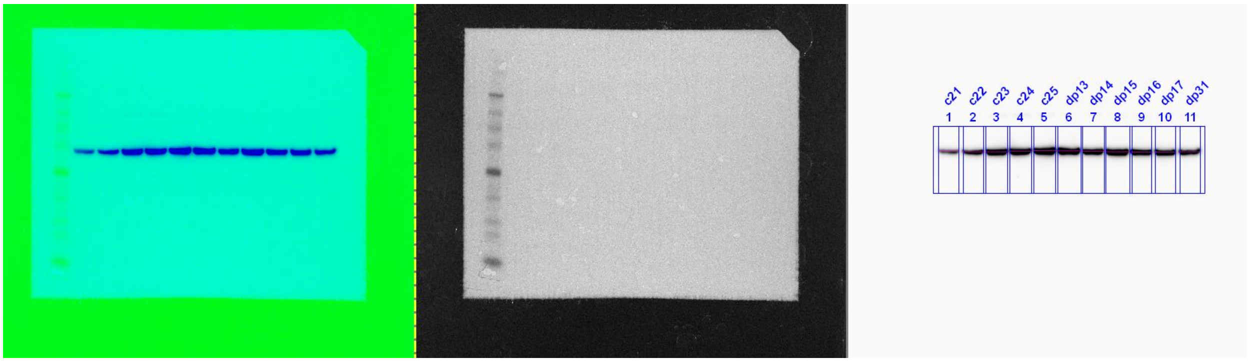

pAMPK protein expression in 30 day rat liver Bands for pAMPK were cropped around 62 kDa according to protein ladder **(a)** and the same membrane was later exposed to actin antibody and cropped around 42 kDa **(b)** Densitometry analysis was performed with the raw data using Image Lab 5.2 software from BioRad

A

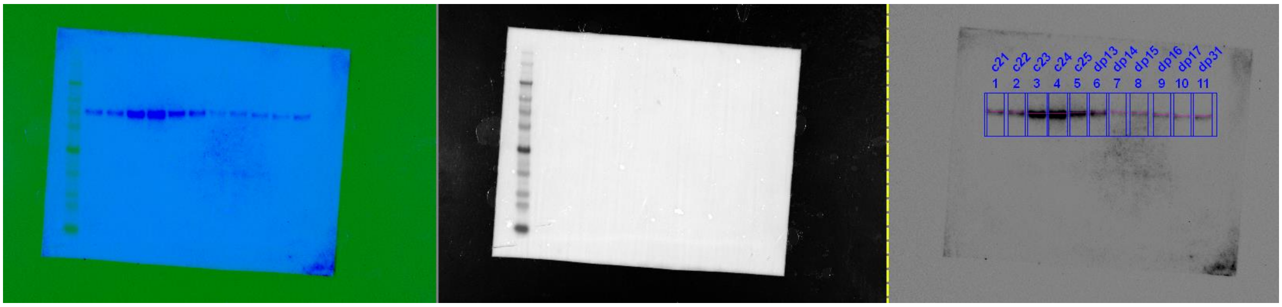

B

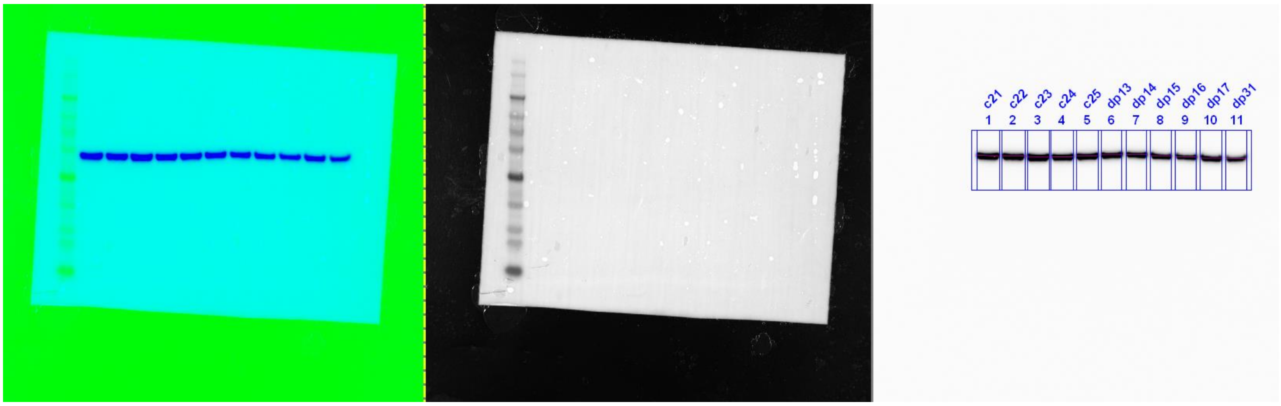

PPARɣ protein expression in 30 day rat liver Bands for PPARɣ were cropped around 54 kDa according to the protein ladder **(a)** and the same membrane was later exposed to actin antibody and cropped around 42 kDa **(b)** Densitometry analysis was performed with the raw data using Image Lab 5.2 software from BioRad
